# Supplementary material for: Design of Cyclic Peptide-Based Nanospheres and the Delivery of siRNA
Source: Int J Mol Sci. 2022 Oct 11;23(20):12071. doi: 10.3390/ijms232012071 (PMC9602810; doi:10.3390/ijms232012071)
Supplement: Supplementary file 1 [file ijms-23-12071-s001.zip › ijms-1915754-supplementary.pdf]

Article

Design of Cyclic Peptide-based Nanospheres and the Delivery of siRNA

Junfeng Ke [1,2], Jingli Zhang [1,2], Wei Luo [3], Junyang Li [1,2], Junqiu Liu [4,*] and Shuwen Guan [1,2,*]

Supplementary data

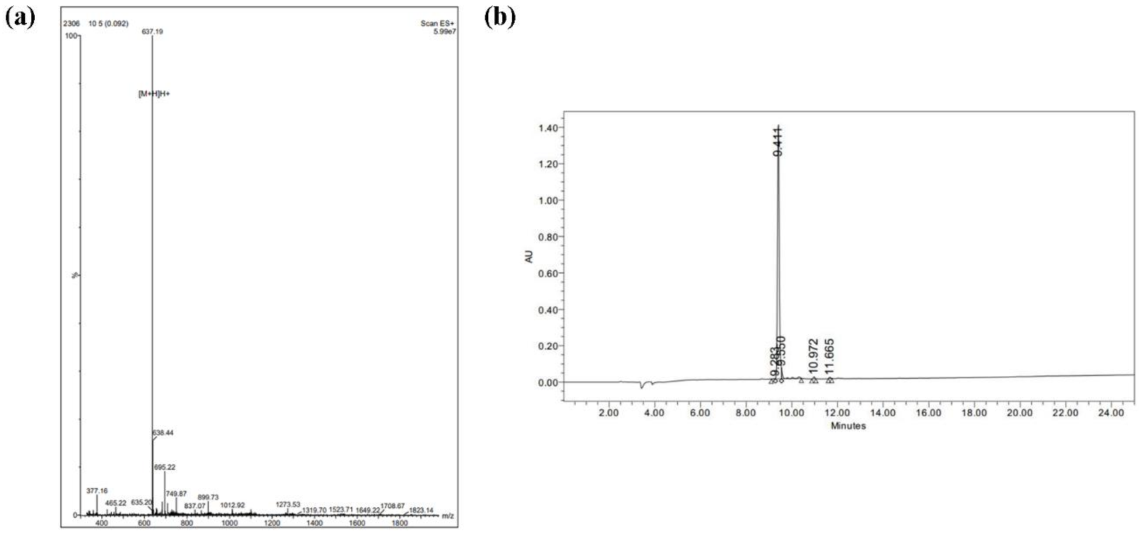

Figure S1. Identification and purification of cyclo-(DP)3. (**a**) Mass spectrometry of cyclo-(DP)3. (**b**) High Performance Liquid Chromatography of cyclo-(DP)3.

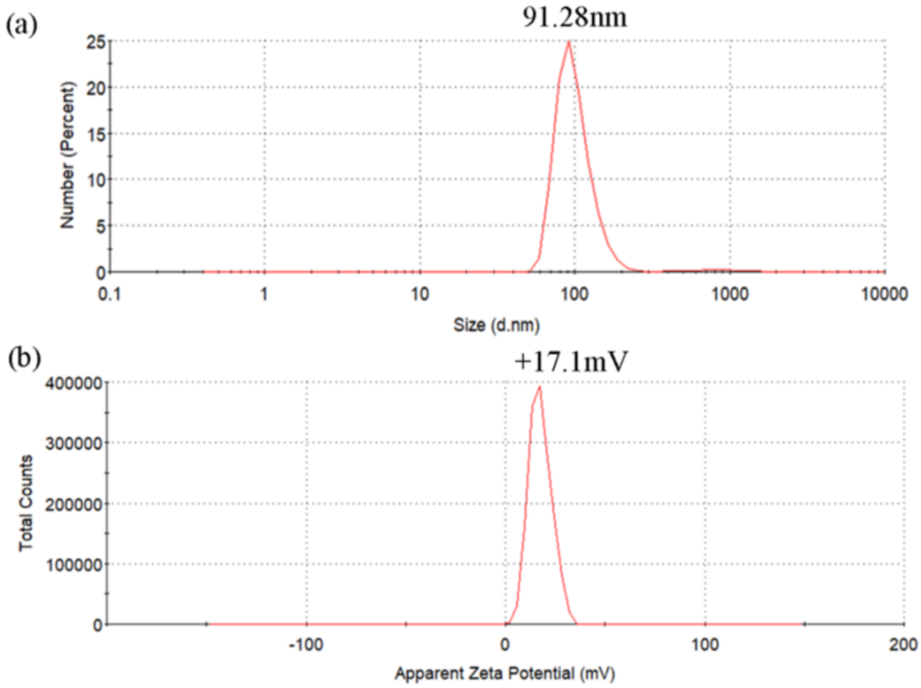

Figure S2. Dynamic Light Scattering (DLS) characterization of (CP)6NS. Particle size distribution (**a**) and surface potential (**b**) of (CP)6NS.

Table S1. Hemolysis rate of 20-500 μg/mL $(CP)_6NS$.

**Samples**	**Concentration (μg/mL)**	**Mean OD value**	**Hemolysis(%)**
Saline	–	0.100 ± 0.0006	0
Distilled water	–	1.439 ± 0.0015	100
	20	0.11 ± 0.0017	0.74
$(CP)_6NS$	200	0.118 ± 0.0023	1.41
	500	0.121 ± 0.0006	1.60

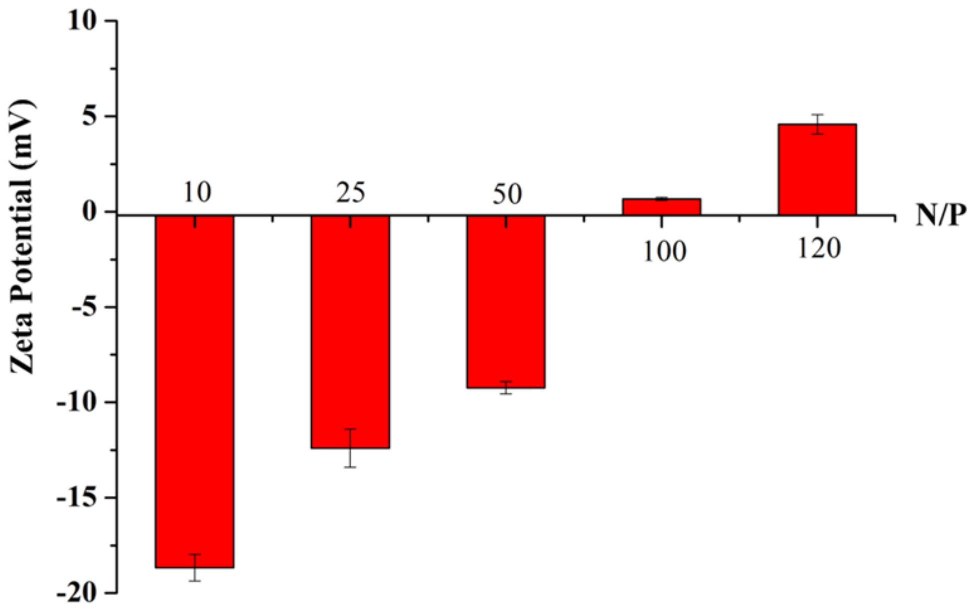

Figure S3. Zeta potential of $(CP)_6NS$-siRNA complexes at different N/P.

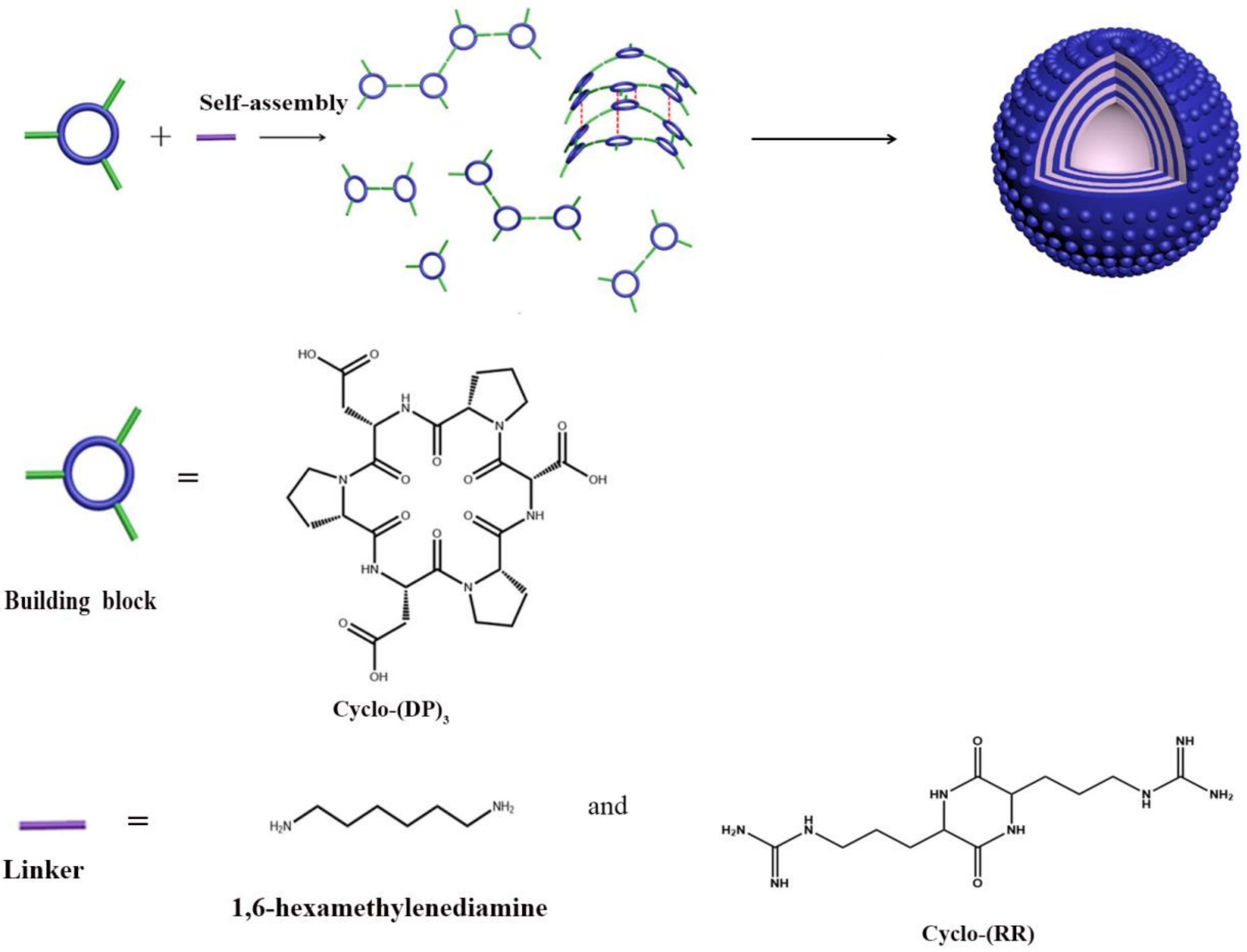

Figure S4. Schematic diagram of self-assembly of cyclic peptide nanospheres.

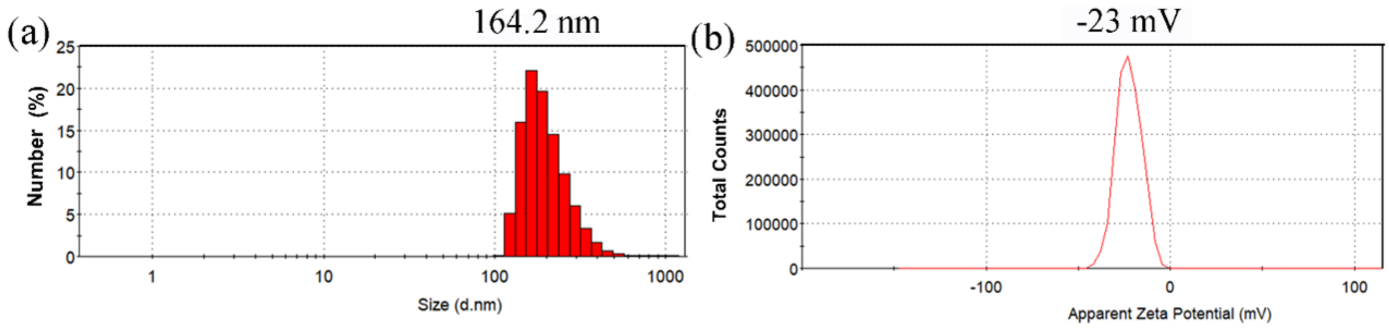

Figure S5. Dynamic Light Scattering (DLS) characterization of covalent assembly of cyclo-(DP)3 and 1,6-hexanediamine. Particle size distribution (**a**) and surface potential (**b**) of assemblies formed by covalent assembly of cyclo-(DP)3 and 1,6-hexanediamine.

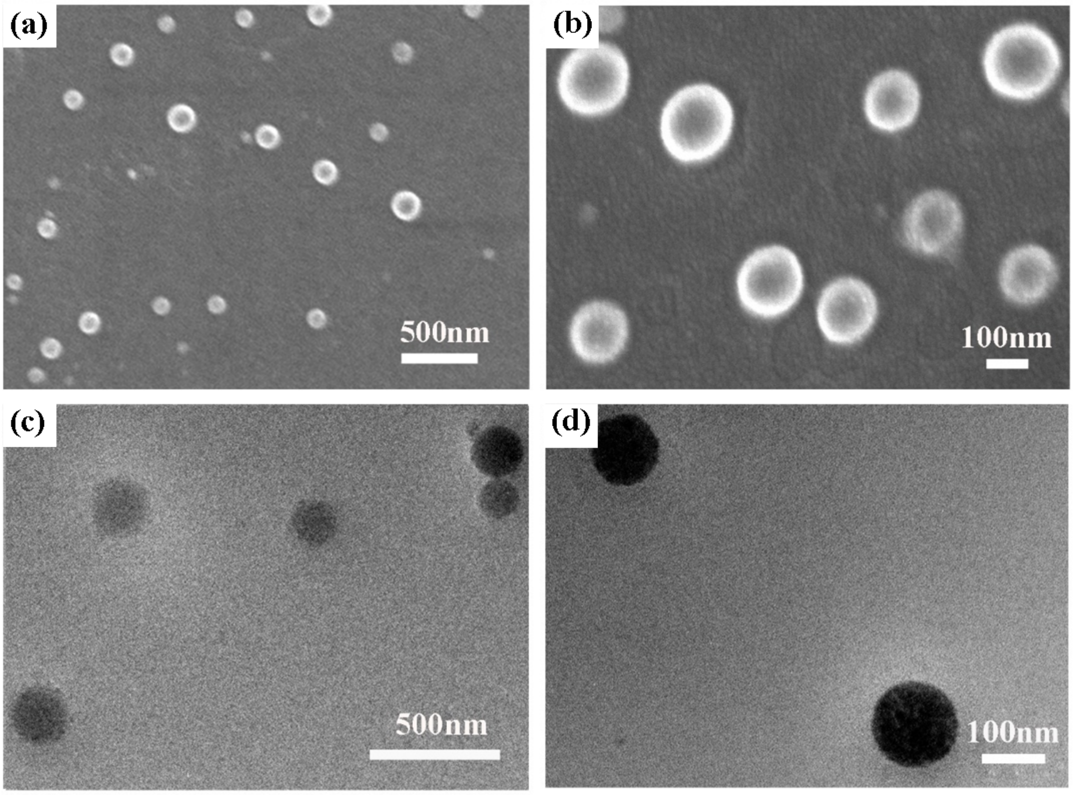

Figure S6. Characterization of covalent assemblies of cyclo-(DP)3 and 1,6-Hexanediamine. (**a, b**) Scanning electron microscope image. (**c, d**) TEM image.

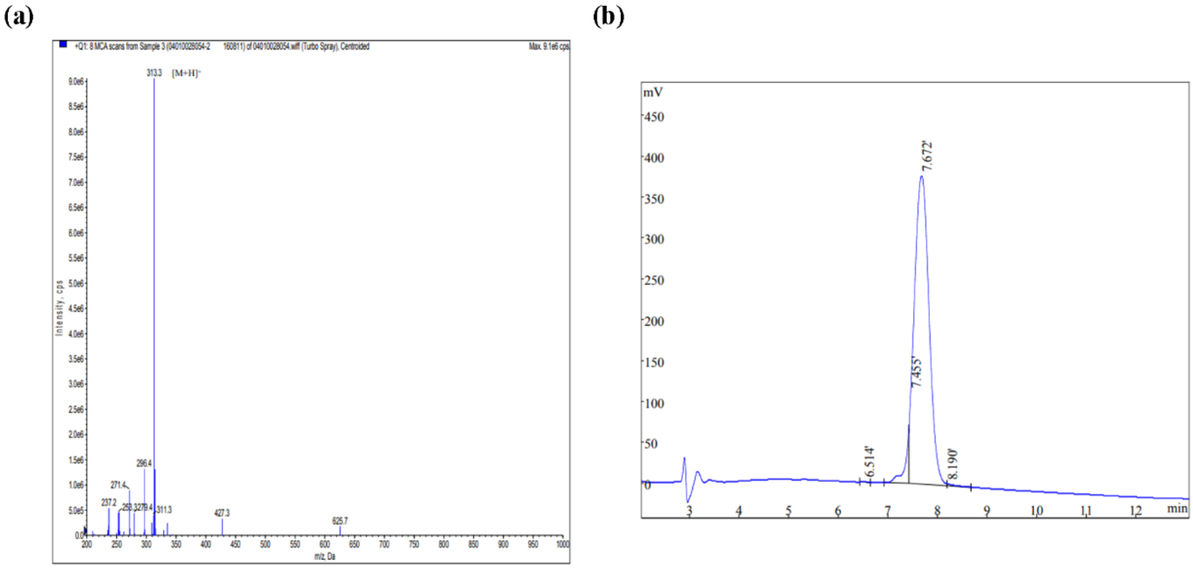

Figure S7. Identification and purification of cyclo-(RR). (**a**) Mass spectrometry of cyclo-(RR). (**b**) High Performance Liquid Chromatography of cyclo-(RR).

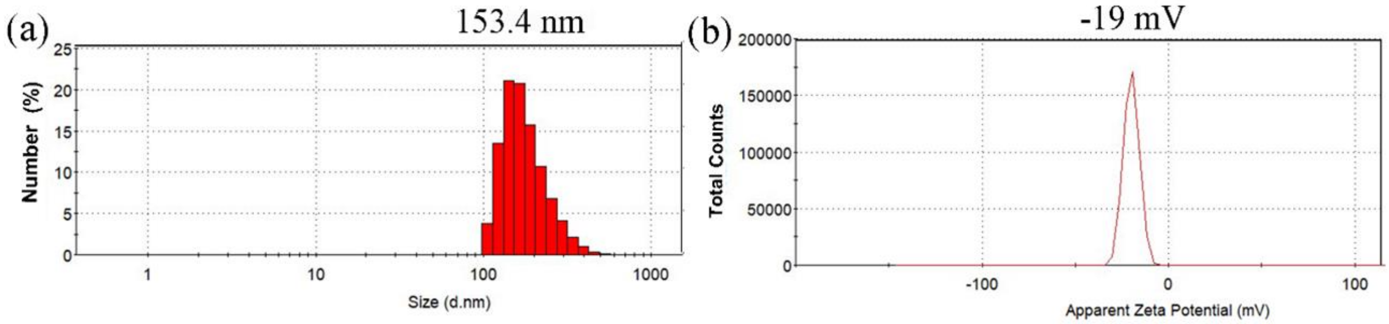

Figure S8. Dynamic Light Scattering (DLS) characterization of covalent assembly of cyclo-(DP)3 and cyclo-(RR). Particle size distribution (**a**) and surface potential (**b**) of assemblies formed by covalent assembly of cyclo-(DP)3 and cyclo-(RR).

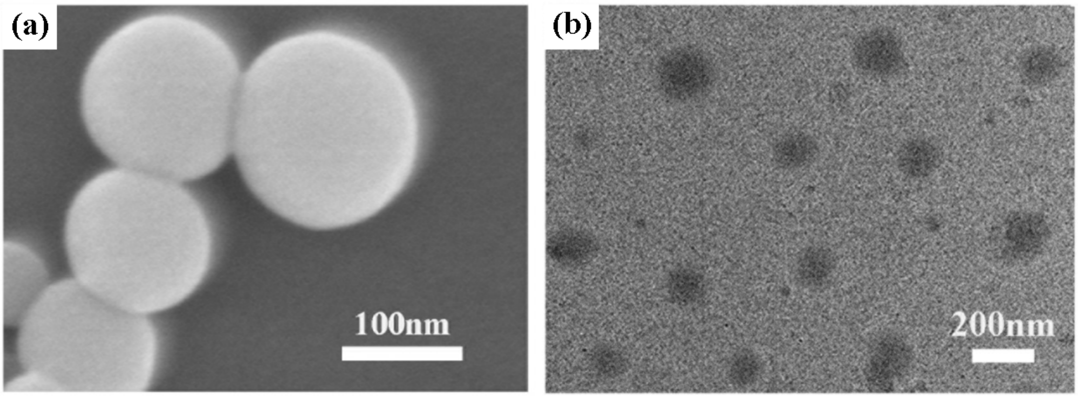

Figure S9. Characterization of covalent assemblies of cyclo-(DP)3 and cyclo-(RR). (**a**) Scanning electron microscope image. (**b**) TEM image.

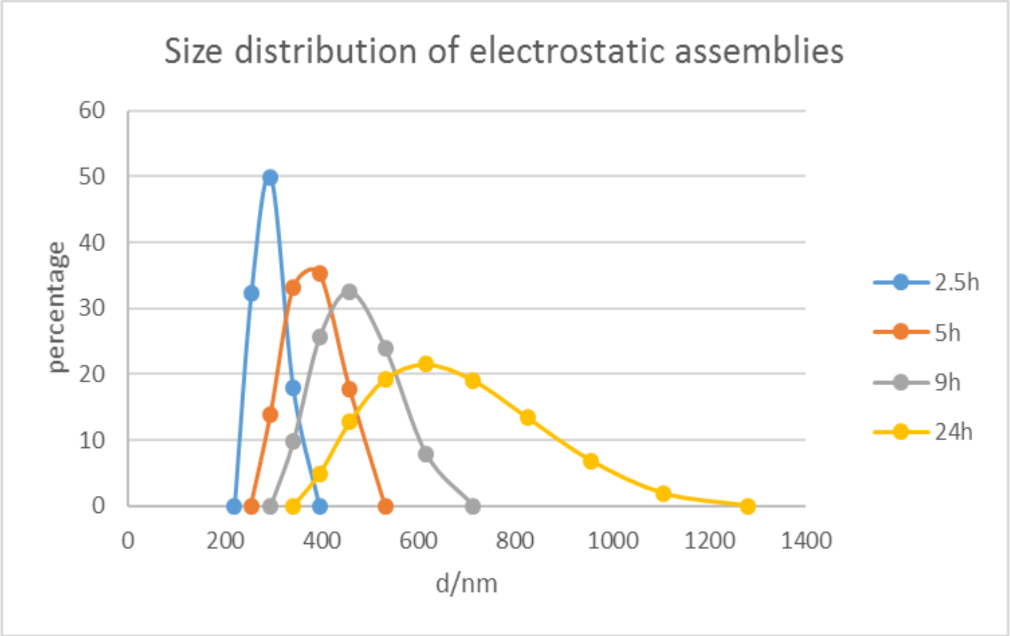

Figure S10. Cyclo-(DP)3 and 1,6-hexanediamine were reacted for different times and then PyBOP was added. DLS characterized size distribution of the electrostatic vesicles at different time.

Table S2. Sequences of primers.

Primer		Sequence
survivin siRNA	FWD	AGCAUUCGUCCGGUUGCGCTT
	REV	GCGCAACCGGACGAAUGCUTT
scramble siRNA	FWD	UUCUCCGAACGUGUCACGUTT
	REV	ACGUGACACGUUCGGAGAATT
FAM-siRNA	FWD	CGUACGCGGAAUACUUCGATT
	REV	UCGAAGUAUUCCGCGUACGTT
survivin	FWD	CCAGATGACGACCCCATAGAG
	REV	TTTGCAATTTTGTTCTTGGCCT
GAPDH	FWD	CCAATGTGTCCGTCGTGGAT
	REV	GAGTTGCTGTTGAAGTCGCA
